# Genetic diversity and antimicrobial resistance of *Salmonella* serotypes recovered throughout the beef production chain and from patients with salmonellosis

**DOI:** 10.7717/peerj.5482

**Published:** 2018-08-23

**Authors:** Mauricio Realpe-Quintero, Jeannette Barba-León, Julia A. Pérez-Montaño, Carlos Pacheco-Gallardo, Delia González-Aguilar, Rosa M. Dominguez-Arias, Elisa Cabrera-Diaz

**Affiliations:** 1 Universidad de Guadalajara, Departamento de Medicina Veterinaria, Centro Universitario de Ciencias Biológicas y Agropecuarias, Zapopan, Jalisco, Mexico; 2 Universidad de Guadalajara, Departamento de Salud Pública, Centro Universitario de Ciencias Biológicas y Agropecuarias, Zapopan, Jalisco, Mexico; 3 Universidad de Guadalajara, Marcelino García Barragán, Departamento de Farmacobiología, Centro Universitario de Ciencias Exactas e Ingenierías, Guadalajara, Jalisco, Mexico; 4 Universidad de Guadalajara, Departamento de Biología Celular y Molecular, Centro Universitario de Ciencias Biológicas y Agropecuarias, Zapopan, Jalisco, Mexico

**Keywords:** Antimicrobial resistance, Serotypes, PFGE, Salmonella, Raw beef

## Abstract

*Salmonella* is one of the major foodborne pathogens worldwide. The antimicrobial resistance (AMR) of this foodborne pathogen has raised a great concern in recent years. Studies on the frequency and characterization of *Salmonella* serotypes can help to improve our knowledge on the epidemiology of this pathogen. The purpose of this study was to compare the serotypes, AMR and genetic profiles of *Salmonella* isolates recovered from raw beef throughout the beef production chain and from human feces associated with clinical cases of salmonellosis. The serotype, AMR and pulsed-field gel electrophoresis profile of 243 *Salmonella enterica* isolates recovered from beef carcasses (*n* = 78), ground beef (*n* = 135), and human feces from clinical cases of salmonellosis (*n* = 30) were compared. Forty-three different *Salmonella* serotypes were identified and regardless of the source, the top five corresponded to Typhimurium, Give, Group B (partially serotyped), Infantis and Anatum. Twelve serotypes from beef carcasses were also found in ground beef, showing their presence throughout the beef production chain.* Salmonella* Typhimurium, Infantis, Anatum and Montevideo were the only serotypes identified in all sample types. Resistance to tetracyclines was the most frequent (41.2%) followed by resistance to aminoglycosides (37%), folate pathway inhibitors (21%), quinolones (20.2%), phenicols (17.1%), penicillins (15.6%) and cephems (7%). Multidrug resistance was observed in 28.8% of the isolates, and those from human feces showed resistance to a larger number of antimicrobials. Great concern arises from the resistance and reduced susceptibility observed to quinolones and cephalosporins because these drugs are the first line of treatment for invasive *Salmonella* infections. Twenty-seven distinct pulse-types were detected among 238 isolates. Clustering analysis for the most frequent serotypes identified groups of isolates with similar AMR profiles. Multidrug resistance spreading throughout the food production chain should be continually monitored and its importance emphasized.

## Introduction

*Salmonella* is one of the major foodborne pathogens worldwide. The World Health Organization (WHO) has measured the global burden of foodborne diseases and estimated that non-typhoidal *Salmonella enterica* accounted for more than 78 million cases of foodborne illnesses worldwide with approximately 59,000 deaths in 2010 (Havelaar et al., 2015; World Health Organization, 2015b). In Mexico, national health authorities reported more than 5 million cases of gastrointestinal infections in 2016, constituting the second cause of morbidity in this country; 77,614 of those (approximately 1.5%) corresponded to cases of salmonellosis (Secretaríade Salud, 2016).

The infections caused by non-typhoidal *Salmonella* are related to the consumption of water and contaminated foods. The pathogen has been isolated from a wide variety of foods, mainly of animal origin (Neto et al., 2010; Painter et al., 2013). Food animals are the principal reservoirs for this pathogen and in consequence, it is frequently isolated from raw meats. The prevalence of *Salmonella* has been reported from 3.1 to 45.2% on pre-eviscerated beef carcasses, and from 0.1 to 7.6% on beef carcasses post-interventions (Hanson et al., 2016). In ground beef, the pathogen’s prevalence was 1.3 and 1.6%, in Canada and the United States (US), respectively (American Meat Science Association, 2015; Sorensen et al., 2002; United States Department of Agriculture, 2014a). However, in countries like Turkey and Senegal, the pathogen was isolated from 21.3 and 87.4% of ground beef samples analyzed (Arslan & Eyi, 2010; Stevens et al., 2006). Similarly, high frequencies of *Salmonella* in raw meats has been reported in Mexico; the pathogen was isolated from 6.4 to 28.6% of beef carcass samples collected at small non-federally inspected abattoirs (Perez-Montano et al., 2012), from 29.9% of raw beef samples collected at retail, and from 56.7 to 71% of ground beef samples obtained from butcher’s shops (Cabrera-Diaz et al., 2013; Martinez-Chavez et al., 2015).

Furthermore, a great concern on the antimicrobial resistance (AMR) of foodborne *Salmonella* isolates has raised in recent years, and according to the World Health Organization, the emergence of multidrug-resistant strains constitutes one of the major threats to global health that compromise the ability to treat bacterial infectious diseases and undermine other advances in health and medicine (World Health Organization, 2015a). The surveillance of AMR among foodborne pathogens does not solve the problem by itself. However, through data collection, the source, emergence and spread of resistant strains, can be tracked and the implementation of actions to promote appropriate use of antimicrobials can be improved.

In consequence, investigations on the characterization of non-typhoidal *Salmonella* strains isolated from foods are important because they can help to improve our knowledge on the epidemiology of this pathogen. The characterization of *Salmonella* isolates from foods, animals and humans often describe their serotypes, AMR and genetic profiles, providing valuable epidemiological information to improve food safety policies. In Mexico, these reports are scarce and limited to a few regions of the country (Nayarit-Ballesteros et al., 2016; Zaidi et al., 2008). The purpose of this study was to compare the serotypes, AMR and genetic profiles of *Salmonella* isolates recovered from raw beef throughout the beef production chain and from human feces associated with clinical cases of salmonellosis.

## Materials and Methods

### Origin of *Salmonella* isolates

The serotypes, AMR patterns and pulsed-field gel electrophoresis (PFGE) profiles of 243 *Salmonella enterica* isolates recovered over a three-year period were compared. The isolates were recovered from three types of samples at locations within an estimated area of 1,000 Km^2^ in Jalisco State, Mexico. The serotypes and antimicrobial resistance profiles of only 213 of these isolates were previously published by our research group (Cabrera-Diaz et al., 2013; Perez-Montano et al., 2012); from these, 78 were isolated from beef carcass samples (coded as C) collected at small abattoirs, and 135 from ground beef samples (coded as G) collected at butcher’s shops. The PFGE profiles of these strains were determined in the present study. In order to compare isolates from food and human origin, 30 *Salmonella* isolates were recovered from human feces (coded as H) associated with clinical cases of diarrhea occurring in health-care institutions; their serotypes, AMR and PFGE profiles were analyzed for this investigation. The H isolates were characterized at the Food Safety Laboratory (University of Guadalajara, Mexico), and kept on tryptic soy agar (TSA, BD Diagnostic Systems, Sparks MD) slants at 4 °C for testing. Each isolate was individually grown in tryptic soy broth (TSB, BD Diagnostic Systems) at 35 °C overnight, then they were streaked on brilliant green sulfa agar (BD Diagnostic Systems); plates were incubated at 35 °C for 24 hours. Typical colonies were biochemically tested and confirmed as *Salmonella* by a multiplex polymerase chain reaction (PCR) assay to detect the presence of *invA* (544 bp) and *fimA* (686 bp) genes, using previously described primers and methods (Perez-Montano et al., 2012). The isolates were sent to the National Laboratory for Diagnosis and Epidemiological Reference (INDRE, Mexico City, Mexico) for serotyping according to the Kauffman-White scheme.

### Antimicrobial susceptibility testing

Antimicrobial susceptibility was tested against ampicillin (10 µg, AMP), gentamicin (10 µg, GEN), tetracycline (30 µg, TET), trimethoprim-sulfamethoxazole (1.25/23.75 µg, SXT), chloramphenicol (30 µg, CHL), ceftriaxone (30 µg, CRO), ciprofloxacin (5 µg, CIP), kanamycin (30 µg, KAN), nalidixic acid (30 µg, NAL), streptomycin (10 µg, STR), and cephalothin (30 µg, CEP). The test was performed according to the disk diffusion method on Mueller-Hinton agar (Clinical Laboratory Standards Institute, 2006) using commercial disks (BBL Microbiology Systems, Cockeysville, MD). Inhibition zones were measured and interpreted according to the Clinical and Laboratory Standards Institute supplement (Clinical Laboratory Standards Institute, 2014). *E. coli* ATCC 25922 was used as quality control. *Salmonella* isolates showing resistance to three or more antimicrobial classes were classified as multidrug resistant (MDR) (CDC, 2018). To determine if the frequency of isolates resistant to at least one antimicrobial class, or the frequency of MDR isolates was affected by the origin of the samples (B, G or H), or by serotype, Chi-square tests were performed using the SPSS v22 software. When significant differences were found, post-hoc tests were conducted using the procedure recommended by Beasley & Schumacker (1995).

### Pulsed-field gel electrophoresis (PFGE)

PFGE was performed for the 243 *Salmonella enterica* isolates according to the standard protocol developed by the Centers for Disease Control and Prevention (CDC, 1996). Briefly, agarose-embedded DNA was digested with 50 U of the enzyme XbaI (Promega, Southampton, UK). DNA restriction fragments were separated by PFGE on 1% SeaKem Gold agarose (Cambrex Bio Science Rockland Inc., Rockland, ME, USA) using 0.5X Tris-Borate-EDTA extended-range buffer (Bio-Rad, Hercules, USA) with recirculation at 14 °C in a CHEF DRIII system (Bio-Rad, Hercules, USA). DNA from *Salmonella* Braenderup H9812 (Kindly provided by Dr. E. Calva from Instituto de Biotecnología, Universidad Nacional Autónoma de México) was digested with restriction endonuclease XbaI, and it was used as a size marker. Pulse times were ramped from 2.2 to 63.8 s over 21 h with an angle of 120° at 6.0 V cm^−1^. Genomic-DNA profiles or ‘pulsetypes’ were manually analyzed. Distinct pulsotypes were assigned to isolates when any differences in electrophoretic mobility were found. Following separation, EtBr staining, and image capture, PFGE banding patterns were visually inspected to detect polymorphic segments. DNA fragments exhibiting sizes above 54.7 S were taken into consideration to define informative characters, based on a side-by-side comparison with the reference *Salmonella* Braenderup H9812 strain, as recommended by the CDC guidelines. DNA fragments showing size variation were coded into a presence/absence matrix to obtain a dataset of informative characters. A total of 27 polymorphic DNA fragments were detected by PFGE and were later used for cluster analysis; 13 out of the 27 fragments were commonly found in *S*. Braenderup and the other 14 fragments were found only among the analyzed isolates, for a total similarity matrix of 27 informative characters and 238 taxa in the final dataset. The PFGE profiles of five isolates were not obtained.

The 238 isolates PFGE firgerprints were organized in a similarity matrix which establishes the presence/absence of such in comparison with a control. After that, they were subject to cluster analysis using the Unweighted Pair Group Method using Arithmetic averages (UPGMA) algorithm as implemented in a web-provided platform (http://genomes.urv.cat/UPGMA) (Garcia-Vallvé & Puigbo, 2002). The Pearson correlation coefficient (r) was selected to estimate similarity as equivalent to parameters used in specialized software for PFGE analysis (Applied Maths, 2011). Detection of DNA fragments in gels was digitally recorded, and subsequently used for visual inspection and manual codification of pulsetypes. No correction for differences in bands intensity was considered.

**Table 1:** Multidrug resistance of *Salmonella* serotypes isolated from raw beef and human feces.

Serotype	Number of isolates (%)									
	*n*	(%)	Beef carcasses^a^ (C)	Ground beef^b^ (G)	Human feces (H)	Number of antimicrobial classes to which isolates showed resistance^c^				
						0	1–2	3–4	5–6	7
Typhimurium	28	(11.5)	14	8	6	4	3	8	11	2
Group B	24	(9.9)	11	13	0	5	2	12	4	1
Give	23	(9.5)	19	4	0	19	3	0	1	0
Infantis	19	(7.8)	8	9	2	7	11	1	0	0
Anatum	17	(7.0)	4	12	1	9	2	3	3	0
Agona	10	(4.1)	0	9	1	3	6	1	0	0
Havana	10	(4.1)	2	8	0	6	2	2	0	0
Group E1	9	(3.7)	3	6	0	4	5	0	0	0
Group E1 monophasic	9	(3.7)	1	8	0	7	2	0	0	0
Derby	7	(2.9)	0	7	0	3	0	3	1	0
Sinstorf	7	(2.9)	1	6	0	6	0	0	1	0
Enteritidis	6	(2.8)	1	0	5	2	4	0	0	0
Muenchen	6	(2.5)	0	1	5	4	1	1	0	0
Panama	6	(2.5)	1	5	0	2	3	0	1	0
Montevideo	5	(2.1)	3	1	1	5	0	0	0	0
Brandenburg	4	(1.7)	0	4	0	1	2	1	0	0
Rissen	4	(1.6)	0	4	0	3	0	0	1	0
Saintpaul	4	(1.6)	0	4	0	1	0	3	0	0
Albany	3	(1.2)	0	2	1	0	2	1	0	0
Bovismorbificans	3	(1.2)	3	0	0	3	0	0	0	0
Bredeney	3	(1.2)	0	2	1	1	2	0	0	0
Muenster	3	(1.2)	2	1	0	3	0	0	0	0
Braenderup	2	(0.8)	0	2	0	2	0	0	0	0
Group C1	3	(1.2)	0	2	1	2	1	0	0	0
Group G2	2	(0.8)	0	2	0	1	0	1	0	0
Kentucky	2	(0.8)	0	2	0	2	0	0	0	0
Lockleaze	2	(0.8)	0	2	0	1	0	1	0	0
Worthington	2	(0.8)	0	2	0	1	1	0	0	0
Group 18	1	(0.4)	0	1	0	0	1	0	0	0
Adelaide	1	(0.4)	0	1	0	0	1	0	0	0
Azteca	1	(0.4)	0	1	0	1	0	0	0	0
Cabbstatt	1	(0.4)	0	1	0	0	1	0	0	0
Duesseldorf	1	(0.4)	0	0	1	1	0	0	0	0
Group G1	1	(0.4)	0	1	0	1	0	0	0	0
Group 2 monophasic	1	(0.4)	0	1	0	1	0	0	0	0
Group B monophasic	1	(0.4)	1	0	0	0	0	1	0	0
Group D	1	(0.4)	0	0	1	1	0	0	0	0
Livingstone	1	(0.4)	1	0	0	1	0	0	0	0
London	1	(0.4)	0	0	1	0	1	0	0	0
Manhattan	1	(0.4)	0	0	1	1	0	0	0	0
Oranienburg	1	(0.4)	1	0	0	1	0	0	0	0
Reading	1	(0.4)	0	1	0	0	0	1	0	0
Urbana	1	(0.4)	0	0	1	0	0	1	0	0
Untypeable	5	(2.1)	2	2	1	1	0	4	0	0
TOTAL	243	100	78	135	30	116	56	45	23	3

**Notes.**

a Perez-Montano et al. (2012).

b Cabrera-Diaz et al. (2013).

c Antimicrobial classes include aminoglycosides, cephems, folate pathway inhibitors, penicillins, phenicols, quinolones and tetracyclines.

## Results

The present investigation consisted of a descriptive study on the diversity and relatedness of 243 *Salmonella enterica* strains isolated from C (*n* = 78), G (*n* = 135) and H (*n* = 30) samples based on their serotype, AMR and PFGE profile. Forty-three different serotypes were identified among 238 *Salmonella* isolates (Table 1) and five strains were untypeable. Regardless of the source, the top five serotypes identified corresponded to *S.* Typhimurium, Group B (partially serotyped), Give, Infantis and Anatum. The larger serotype diversity was observed among isolates from ground beef (G samples) with 33 different serotypes identified from 135 isolates; *S.* Group B, Anatum and Infantis were the most common in this type of samples. From beef carcasses (C samples), 17 serotypes out of 78 isolates were identified and the most frequent were *S.* Give, Typhimurium and Group B. Among 30 isolates recovered from human feces (H samples), 15 serotypes were identified and the most common were *S.* Typhimurium, Enteritidis and Muenchen.

Twelve of the serotypes found on C were also present on G samples, showing their presence throughout the beef production chain. Some other serotypes were probably introduced into ground beef at the butcher’s shops because fabrication of ground beef, ground pork, and sometimes ground chicken, using the same grinder during the same working day was previously identified as a common practice in these establishments (Cabrera-Diaz et al., 2013). Ten out of the 15 serotypes identified from H, were also present either on C or G samples. *Salmonella* Typhimurium, Infantis, Anatum and Montevideo were identified on H, C and G; on the contrary, *S.* Enteritidis and Muenchen were frequent on H, but not on C or G samples.

In addition to serotyping, the AMR profile of the 243 *Salmonella* isolates was compared. Resistance to at least one antimicrobial class was present in 52% (*n* = 127) of the isolates. In general, resistance to tetracyclines (TET) was the most frequent as it was present in 41.2% of the isolates (Table 2), followed by resistance to aminoglycosides (KAN, STR and GEN, 37%), folate pathway inhibitors (SXT, 21%), quinolones (NAL and CIP, 20.2%.), phenicols (CHL, 17.7%), penicillins (AMP, 15.6%), and cephems (CEP and CRO, 7%). No significant differences were observed on the frequency of resistant isolates to each antimicrobial according to their origin (*p* > 0.05).

**Table 2:** Antimicrobial resistance (R) and intermediate susceptibility (I) of *Salmonella* isolates from raw beef and human feces.

Antimicrobial class	Proportion of isolates expressed as %							
	Beef carcasses (*n* = 78)		Ground beefr (*n* = 135)		Human feces^a^ (*n* = 30)		Total R (*n* = 243)	Total I (*n* = 243)
	R^*b*^	I^*c*^	R	I	R	I		
Tetracyclines (TET)	46.2	0.0	40.7	0.0	30.0	3.3	41.2	0.4
Aminoglycosides (KAN, STR, GEN)	42.3	21.8^**^	36.3	5.2	26.7	10.0	37.0	11.1
Folate pathway inhibitors (SXT)	21.8	2.6	20.7	3.0	20.0	3.3	21.0	2.9
Quinolones (NAL, CIP)	19.2	35.9	20.0	43.7	23.3	20.0	20.2	38.3
Phenicols (CHL)	23.1	2.6	14.8	6.7	16.7	0.0	17.7	4.5
Penicillins (AMP)	9.0	0.0	17.8	0.0	23.3	0.0	15.6	0.0
Cephems (CEP, CRO)	3.8	7.7	6.7	22.2^**^	16.7	6.7	7.0	15.6

**Notes.**

a Fecal samples from human clinical cases of diarrhea.

b R = proportion of isolates (%) showing resistance to the at least one antibiotic of the antimicrobial class.

c I = proportion of isolates (%) showing intermediate susceptibility to at least one antibiotic of the antimicrobial class.

TET tetracycline KAN kanamycin STR streptomycin GEN gentamicin SXT trimethoprim-sulfamethoxazole NAL nalidixic acid CIP ciprofloxacin CHL chloramphenicol AMP ampicillin CEP cephalothin CRO ceftriaxone

** The proportion of isolates showing intermediate susceptibility was significantly different for that type of sample (*p* < 0.05).

Reduced susceptibility to quinolones was frequent on isolates from all types of samples (Table 2). Furthermore, 63.8% of isolates resistant to NAL also showed intermediate susceptibility to CIP (Table 2), most of them corresponding to serotype Typhimurium. The frequency of isolates showing intermediate susceptibility to cephems was significantly higher on those recovered from G samples (*p* < 0.05). For aminoglycosides, the frequency of intermediate-susceptible isolates was significantly higher on those obtained from C samples (*p* < 0.05).

MDR was present in 70 (28.8%) of 243 isolates (Table 3) and no differences were observed according to their origin (*p* > 0.05). Three isolates (1.2%) showed resistance to the seven classes of antimicrobials tested. *Salmonella* Typhimurium was the most common MDR serotype followed by Group B, Anatum and Derby (Table 1). Two *Salmonella* Typhimurium isolates recovered from H samples were resistant to 10 antimicrobials of seven different classes (Table 3). The proportion of MDR isolates was significantly higher (*p* < 0.05) for serotypes Typhimurium (75%) and Group B (70.9%).

**Table 3:** Multidrug-resistance of *Salmonella* isolates recovered from human feces and raw beef.

Origin of isolates	No. (%) of isolates resistant to the indicated number of antimicrobial classes					
	3	4	5	6	7	Total MDR^a^
Beef carcasses (*n* = 78)	6 (7.7)	13 (16.7)	6 (7.7)	0 (0.0)	1 (1.3)	26 (33.4)
Ground beef (*n* = 135)	10 (7.4)	12 (8.9)	13 (9.6)	3 (2.2)	0 (0.0)	38 (28.1)
Human feces (*n* = 30)	2 (6.7)	1 (3.3)	0 (0.0)	1 (3.3)	2 (6.7)	6 (20.0)
*Total* (*n* = 243)	*18(7.4)*	*26 (10.7)*	*19 (7.8)*	*4 (1.7)*	*3 (1.2)*	*70 *(28.8)**
Top 5 serotypes^b^						
Typhimurium (*n* = 28)	1 (3.6)	7 (25.0)	10 (35.7)	1 (3.6)	2 (7.1)	21 (75.0)^**^
Group B^c^ (*n* = 24)	7 (29.2)	5 (20.8)	3 (12.5)	1 (4.2)	1 (4.2)	17 (70.9)^**^
Give (*n* = 23)	0 (0.0)	0 (0.0)	1 (4.3)	0 (0.0)	0 (0.0)	1 (4.3)
Infantis (*n* = 19)	0 (0.0)	1 (5.3)	0 (0.0)	0 (0.0)	0 (0.0)	1 (5.3)
Anatum (*n* = 17)	1 (5.9)	2 (11.8)	1 (5.9)	2 (11.8)	0 (0.0)	6 (35.4)

**Notes.**

a MDR = Multidrug-resistant isolates are those that showed resistance to at least one antibiotic in three or more antibiotic classes tested: penicillins, tetracyclines, quinolones, folate pathway inhibitors, cephems, aminoglycosides or phenicols.

b The five most common *Salmonella* serotypes isolated from all sources.

c Partially serotyped isolates.

** The proportion of isolates showing MDR is significantly different (*p* < 0.05).

Thirteen isolates had MDR phenotypes that included resistance to CIP, and 17 isolates showed MDR phenotypes with resistance to at least one cephalosporin (Table 2). Ten isolates showed the resistant phenotype ACSSuT (resistance to AMP, CHL, STR, SXT and TET), three of them were recovered from H samples (*S*. Typhimurium), and seven isolates from C and G samples (*S.* Anatum, Panama, Sinstorf, and Group B).

In order to compare genetic relatedness among isolates belonging to serotypes identified in the three types of samples (H, G, and C), four of the most common serotypes were selected (*S.* Typhimurium, Infantis, Anatum and Montevideo) and UPGMA clustering analysis was conducted on the final dataset based on the pulse-types. Out of the total 238 PFGE pulse-types identified, 28 corresponded to serotype Typhimurium, 19 to Infantis, 15 to Anatum and five to Montevideo, and they were observed to cluster in good agreement to their AMR profiles. Two isolates of *S.* Anatum were untypeable by PFGE.

**Figure 1:**
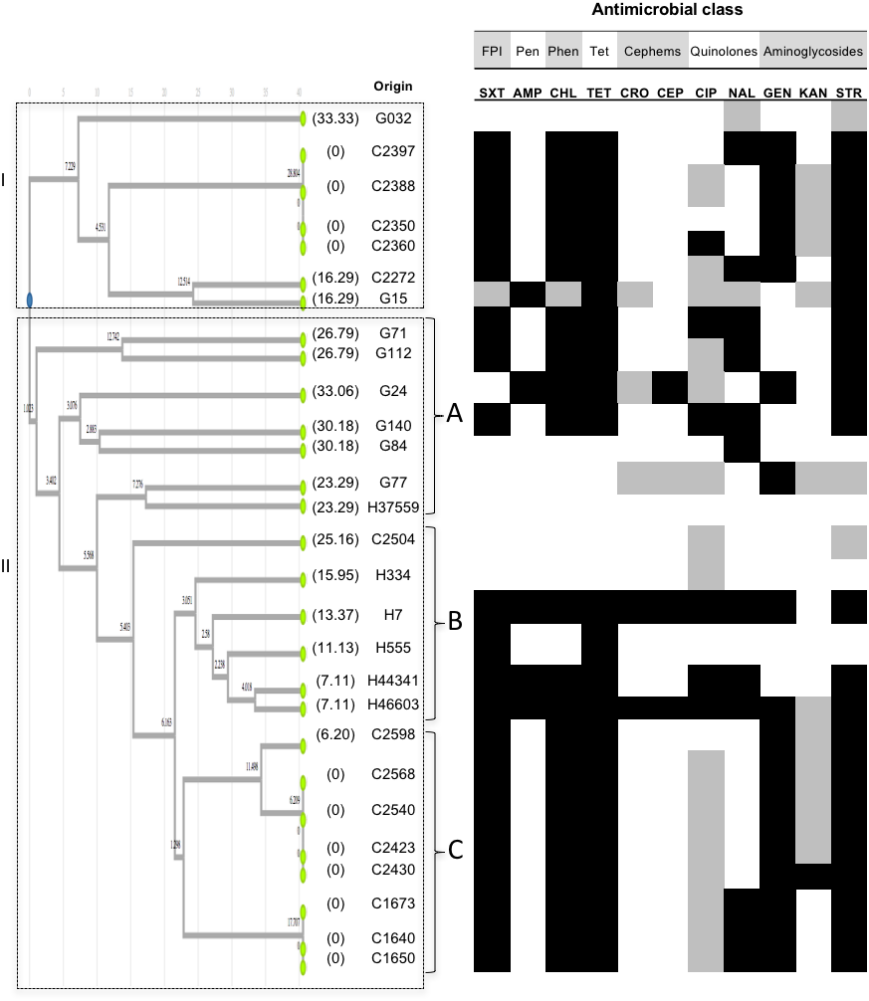
UPGMA clustering analysis Typhimurium. UPGMA clustering analysis of *Salmonella* Typhimurium based on comparison side-by-side of PFGE pulse-types to the reference strain *Salmonella* Braenderup H9812. A dataset of 27 informative characters (presence/absence) exhibiting distinct DNA banding for 28 isolates was used to analyze genetic relatedness. Dendrogram illustrates the UPGMA clustering according to distance metrics based on the r-Pearson correlation coefficient. The corresponding AMR profiles (see text for details) are shown for each isolate. White, gray or black boxes indicate susceptibility, intermediate susceptibility, or resistance to the antimicrobial, respectively. The origin of isolates is represented by letters, where G corresponded to ground beef, C to beef carcasses, and H to human feces. Only serologically confirmed *S.* Typhimurium** isolates were used for the analysis. Values in parentheses represent genetic distances per branch and per leave inside the tree. No cut-off value was defined; clustering was based on total differences found. FPI: folate pathway inhibitors. Pen: penicillins. Phen: phenicols. Tet: tetracyclines.

*Salmonella* Typhimurium, the most common serotype, showed multidrug-resistance profiles that were widely found on isolates from different types of samples (Fig. 1). Twenty-eight *S*. Typhimurium isolates recovered from C (14 isolates), G (eight isolates) and H (six isolates) samples were analyzed by PFGE and UPGMA and two major monophyletic clusters were identified (Fig. 1). For cluster I, seven isolates from C (5 isolates) and G (2 isolates) samples were identified with distances between 0 and 33.33% and assigned to subgroup I. None of the isolates from H samples clustered in this subgroup, and isolates shared resistance to TET, SXT, STR, GEN, and CHL. Within this cluster, four isolates from C samples clustered together showing no genetic differences, which was in partial agreement with their AMR profiles. Cluster II for *S.* Typhimurium included 21 isolates showing genetic distances in the range 0 to 33.06%. Three subgroups were identified in this cluster: subgroup II-A contained seven isolates, six from G, and one isolate from H samples, which was susceptible to the antimicrobial classes tested (Fig. 1). Differences in AMR profiles were observed in subgroup II-A, which was also the subgroup showing the larger genetic distances. Subgroup II-B included five isolates from H and one isolate from C samples clustering together, three of the isolates from H samples showed resistance to 6 of the antimicrobial classes tested, in agreement with the UPGMA clustering based on pulse-types. Subgroup II-C only contained eight isolates from C samples and shared resistance to similar antimicrobial classes as noticed in cluster I.

**Figure 2:**
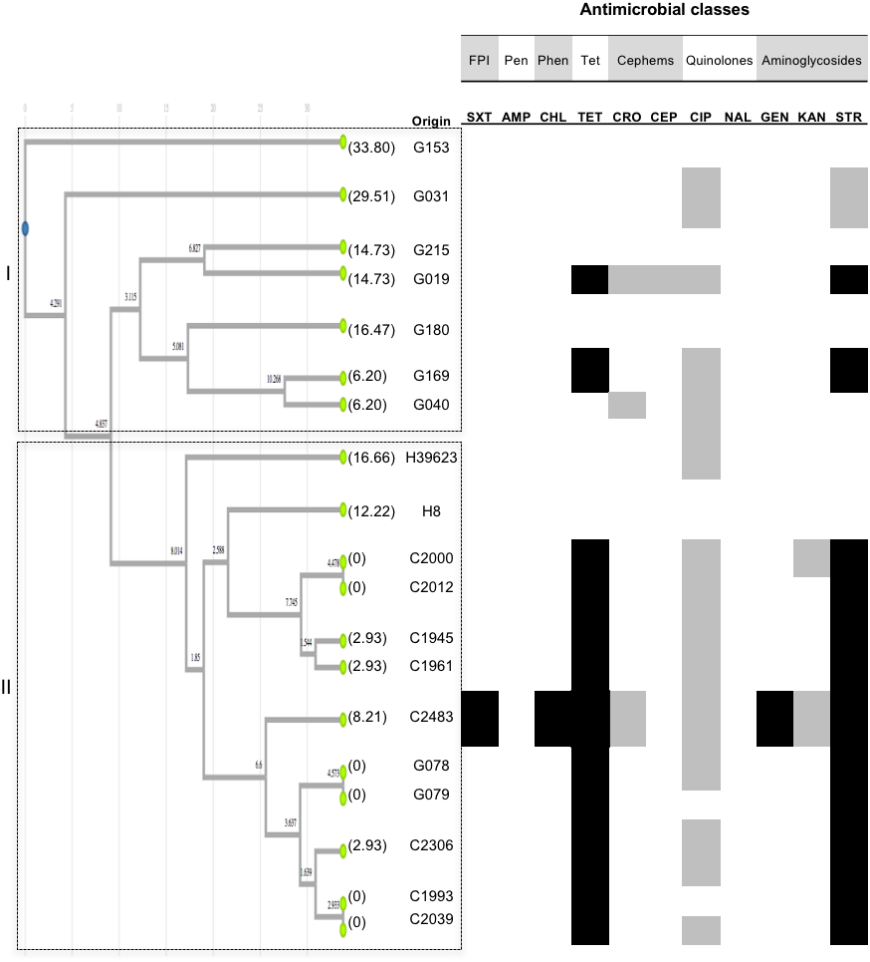
UPGMA clustering analysis Infantis. UPGMA clustering analysis of *Salmonella* Infantis based on comparison side-by-side of PFGE pulse-types to the reference strain *Salmonella* Braenderup H9812. A dataset of 27 informative characters (presence/absence) exhibiting distinct DNA banding for 19 isolates was used to analyze genetic relatedness. Dendrogram illustrates the UPGMA clustering according to distance metrics based on the r-Pearson correlation coefficient. The corresponding AMR profiles (see text for details) are shown for each isolate. White, gray or black boxes indicate susceptibility, intermediate susceptibility, or resistance to the antimicrobial, respectively. The origin of isolates is represented by letters, where G corresponded to ground beef, C to beef carcasses, and H to human feces. Only serologically confirmed *S.* Infantis** isolates were used for the analysis. Values in parentheses represent genetic distances per branch and per leave inside the tree. No cut-off value was defined; clustering was based on total differences found. FPI: folate pathway inhibitors. Pen: penicillins. Phen: phenicols. Tet: tetracyclines.

*Salmonella* Infantis was the second more common serotype identified among the isolates recovered from all types of samples (Table 1), with strains from C (eight isolates), G, (nine isolates) and H (two isolates) samples. The PFGE and UPGMA analysis showed two clusters (Fig. 2). Cluster I with genetic distances from 6.2 to 33.8%, and low similarity among AMR profiles. This cluster only included isolates from G samples. Cluster II was monophyletic and showed a good agreement regarding the resistance phenotype for TET and STR, and intermediate resistance to CIP. Two isolates from H samples were identified in this cluster showing low genetic similarity between them and sharing susceptibility to most of the antimicrobials tested. Two isolates from G samples clustered together within this group, and shared high genetic similarity and AMR profiles between them. Isolates in cluster II exhibited genetic distances in the range 0 to 16.66%.

**Figure 3:**
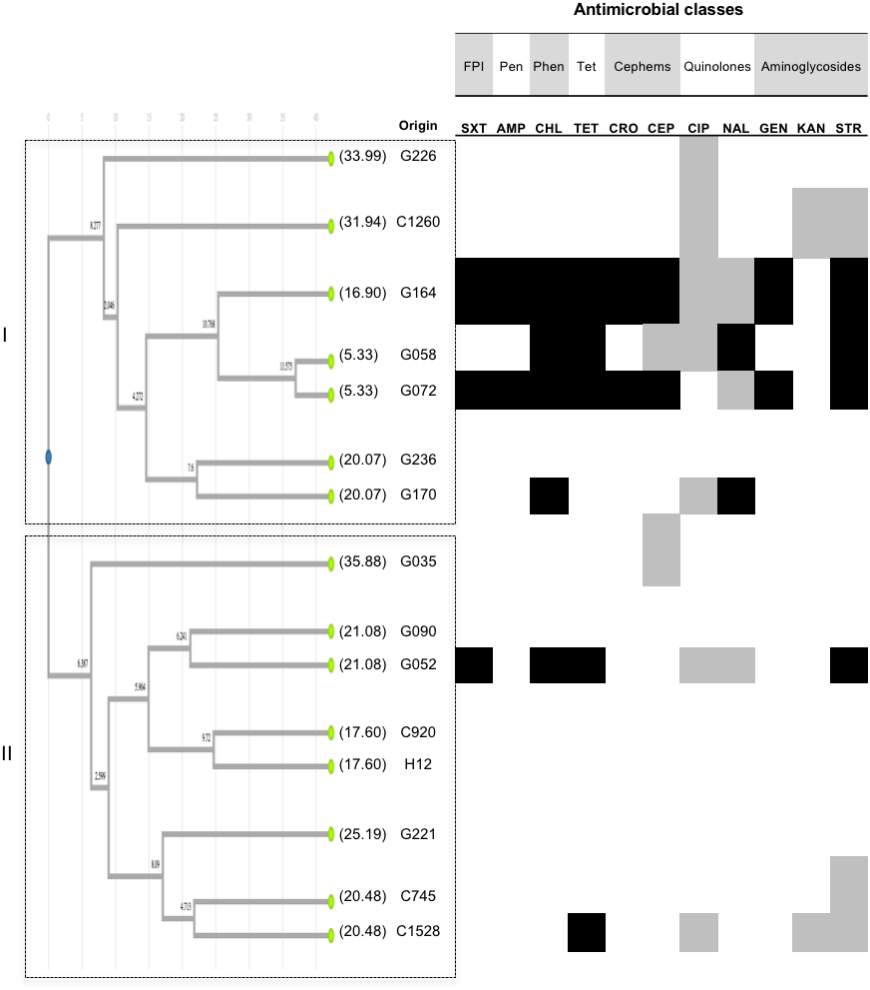
UPGMA clustering analysis Anatum. UPGMA clustering analysis of *Salmonella* Anatum based on comparison side-by-side of PFGE pulse-types to the reference strain *Salmonella* Braenderup H9812. A dataset of 27 informative characters (presence/absence) exhibiting distinct DNA banding for 15 isolates was used to analyze genetic relatedness. Dendrogram illustrates the UPGMA clustering according to distance metrics based on the r-Pearson correlation coefficient. The corresponding AMR profiles (see text for details) are shown for each isolate. White, gray or black boxes indicate susceptibility, intermediate susceptibility, or resistance to the antimicrobial, respectively. The origin of isolates is represented by letters, where G correspond to ground beef, C to beef carcasses, and H to human feces. Only serologically confirmed *S.* Anatum** isolates were used for the analysis. Values in parentheses represent genetic distances per branch and per leave inside the tree. No cut-off value was defined, clustering was based on total differences found. FPI: folate pathway inhibitors. Pen: penicillins. Phen: phenicols. Tet: tetracyclines.

*Salmonella* Anatum was recovered from C (four isolates), G (12 isolates) and H (one isolate) samples (Table 1). PFGE and UPGMA analysis identified two groups. Cluster I comprise six isolates from G and one from C samples (Fig. 3). Among these, low genetic similarity was observed and only two isolates shared similar AMR profiles, showing resistance to eight of the eleven antimicrobials tested belonging to six different classes. Cluster II consisted of eight isolates from G (four isolates), C (three isolates), and H (one isolate) samples. The isolate from a human sample showed higher genetic relatedness to isolates from G and C, and sensitivity to all antimicrobials tested. Isolates in cluster II exhibited genetic distances in the range 0 to 16.66% (Fig. 3). In *S*. Anatum isolates, genetic distances were from 5.33 to 35.88%, which is in agreement to the high variability observed on their AMR phenotypes.

*Salmonella* Montevideo isolates from C (three isolates), G (one isolate) and H (one isolate) samples were compared (Table 1); four isolates showed high genetic relatedness in the range 12.85 to 20.07%. However, the low number of strains do not allow to reach conclusions. In contrast to other serotypes, all *S*. Montevideo strains showed susceptibility to most of the antimicrobials tested. The only *Salmonella* Montevideo isolate that was recovered from human feces did not cluster with other isolates, showing a genetic distance of 37.07%.

## Discussion

*Salmonella* Typhimurium has been reported as one of the principal serotypes isolated from raw beef and responsible for human infections in Mexico and other countries (Zaidi et al., 2008). In the present investigation, this serotype was also the most frequently isolated from all sample types but it was not possible to establish a direct link between the isolates analyzed from raw beef and those obtained from human feces because they were not related in space and time. *Salmonella* strains from C and G samples were obtained previously to those recovered from H and no intent to investigate beef consumption patterns among patients was done. In addition, the number of isolates obtained from H samples was small and may not reflect the actual distribution of *Salmonella* serotypes in patients with a gastrointestinal disease who consume beef in this area of Mexico. Despite these limitations, our study provides valuable data to understand the distribution and diversity of *Salmonella* in this geographical area. Within three years of sampling our data of 43 total serotypes showed the occurrence of 38 different serotypes on C and G samples, and five specific for the H samples, representing a high diversity of *S. enterica* in raw beef that could be affected by inappropriate manufacturing and handling practices at abattoirs and butcher’s shops as previously reported (Martinez-Chavez et al., 2015; Perez-Montano et al., 2012).

A few studies available from Mexico have also demonstrated a large diversity of *Salmonella* serotypes and similar distribution. Gutierrez-Cogco et al. (2000), reported that *S.* Typhimurium, Enteritidis, Derby, Agona and Anatum were the most frequent serotypes recovered from human and non-human sources between 1972 and 1999. Zaidi et al. (2008) found substantial diversity of *Salmonella* serotypes (56 different serotypes) in human and cattle feces, and raw beef samples collected at four States of Mexico, which represent 17% of the national territory; *Salmonella* Typhimurium and Enteritidis were the most commonly found in human feces, while Anatum, Meleagridis, Agona and Typhimurium were most frequent in cattle feces and raw beef samples. In other countries like the US, *Salmonella* Agona, Infantis, Uganda, Anatum and Dublin were the top five serotypes isolated from beef carcasses in 2011, while *S.* Montevideo, Dublin, Cerro, Newport and Muenchen were the top five from ground beef in 2014 (United States Department of Agriculture, 2014a; United States Department of Agriculture, 2014b). In 2013, *S.* Enteritidis, Typhimurium and Newport were the most commonly associated to human infections in that country (CDC, 2016). Despite the geographical differences, our findings show certain similarities with those reports, as we also observed a large diversity of *Salmonella* and we also found *S.* Typhimurium and Anatum among the most frequent serotypes. *Salmonella* Enteritidis, Derby and Agona were not commonly observed in our study probably because most of the analyzed isolates were recovered from beef but not from other types of foods.

The AMR of human pathogens including *Salmonella*, is one of the major threats to global health that compromise the ability to treat infectious diseases and undermine other advances in health and medicine (World Health Organization, 2015a). The large proportion of isolates showing resistance to tetracycline that was observed in our study is probably a consequence of the long history of use of this antibiotic in human therapy and in food animals to treat a wide variety of Gram-positive and Gram-negative bacterial infections since the 1950s. In addition to their effectiveness against traditional bacteria, tetracyclines have also been used to treat infections caused by chlamydiae, mycoplasmas, rickettsiae, protozoan parasites and a variety of noninfectious conditions (Roberts, 2003). Tetracyclines are some of the cheapest classes of antibiotics available today, making them attractive for their use in developing countries, such as Mexico, where health care budgets are limited. These compounds are also commonly used as growth promoters in the main food animal species, and although this usage has been banned in the European Community (De Briyne et al., 2014), it is still legal in many other countries, including Mexico. Animals treated with tetracyclines excrete the drug residues in their feces which can be used as manure for soil amendment and contribute to accumulate antibiotic residues within the soil (Kummerer, 2004). As a result, resistance to tetracyclines is widespread and can be detected in *Salmonella* strains from environmental sources. A survey showed that 60% of *S.* Typhimurium strains isolated from irrigation water in Mexico were resistant to this antimicrobial (Cuevas et al., 2009).

We found a large proportion of isolates with resistance and decreased susceptibility to quinolones, and our findings are in agreement with those reported for *Salmonella* strains from food animals in other countries (Tamang et al., 2011). The resistance or reduced susceptibility to quinolones present in *Salmonella* raise great concern because fluoroquinolones are the first line therapy in systemic salmonellosis. *Salmonella* strains with increased levels of resistance to fluoroquinolones, have been associated with treatment failures (Sjolund-Karlsson et al., 2014).

Regarding cephems, we found that 7% of the isolates were resistant and 15.6% had reduced susceptibility to these compounds, and similar findings have been previously reported in other countries. In the US, 2.4% of *Salmonella* isolates from cattle (97 out of 3,984) showed decreased susceptibility to this drug (Frye et al., 2008). Food animals are considered important reservoirs of CRO-resistant *Salmonella* that cause human illness in that country (Iwamoto et al., 2017). Our findings on resistance or reduced susceptibility to cephalosporins also represent an important concern for public health because these drugs are important for treating invasive *Salmonella* infections, especially in children, among whom fluoroquinolones should be avoided, and also because CRO is the first line of treatment in the presence of quinolone resistance or decreased susceptibility to CIP (Crump et al., 2015). A study carried out in Oregon State (US) demonstrated that the odds of acquiring a non-typhoid *Salmonella* infection caused by a strain resistant to AMP, CRO, CIP, GEN, or SXT, increased significantly each year and patients were more likely to require hospitalization (Barlow et al., 2014). According to this report, resistance to clinically important antimicrobial drugs will increase in that state by 13% each year, and severe illnesses, hospitalizations, and deaths, along with higher economic costs are expected to increase as well.

Moreover, we found that almost 29% of the isolates presented multidrug resistance (MDR) and a significantly larger proportion of MDR isolates was observed among *Salmonella* Typhimurium and *S.* Group B (*p* < 0.05). *Salmonella* infections caused by MDR phenotypes are more likely to result in treatment failure and adverse health outcomes (Mollenkopf et al., 2017).

The WHO Global Action Plan on AMR that was endorsed at the Sixty-Eight World Health Assembly made a call to improve awareness and understanding of AMR, and to strengthen knowledge through surveillance and research as strategic objectives (World Health Organization, 2015a). Important recommendations from the WHO to the agricultural sector include the use of antibiotics to treat infectious diseases under veterinary supervision, and the implementation of international standards for the responsible use of antimicrobials (World Health Organization, 2012). We are aware that surveillance by itself does not reduce the problem; however, data collection helps to track the emergence and spread of resistant strains, promote awareness, and provide information for action to promote an appropriate use of antimicrobials.

The genetic characterization of *Salmonella* isolates through UPGMA together with AMR profiling allows detecting spread of resistance along the food production chain. The finding of similar serotypes on the three types of samples support the observation that some *Salmonella* serotypes are widespread throughout the beef production chain but a high genetic and phenotypic AMR variability was observed intra-serotypes. This could suggest phenotypical changes in the pathogen possibly related to the usage of antimicrobial substances as therapeutic agents and growth promoters in cattle (Mathew, Cissell & Liamthong, 2007; World Health Organization, 2015b). In Greece, *Salmonella* Infantis was also reported along the food production chain, finding a high genetic heterogeneity by PFGE analysis as well (Papadopoulos et al., 2017). When we analyzed the *Salmonella* isolates at genetic level, we found that the UPGMA clustering analysis showed genetic relatedness intra-serotypes, and some patterns were common in the three types of samples (H, G, and C). A good agreement between AMR and pulse-types were found, which could suggest that both analyses provide evidence of phenotypic features underlying genetic variation valuable for adaptation to the selective pressure caused by the use of antimicrobials. As noticed in *S.* Typhimurium, and partially in *S*. Infantis and *S*. Anatum (Figs. 1 to 3), pulse-types allowed isolates with almost identical AMR profiles to cluster together and this was also noticed for *S.* Montevideo. Susceptibility to specific antimicrobials was also noticed to match with clustering of similar isolates based on pulse-types. Altogether, *S.* Typhimurium (Fig. 1) showed that subgrouping coincided with the source of the isolates, and a good correlation of the UPGMA clustering and AMR profiles. This could be due to the larger number of isolates tested for this serotype. We found isolates sharing resistance against AMP, TET, SXT, CIP, STR and CHL, and an additional evaluation is needed to characterize the virulence islands at a molecular level, to determine if they could be a harboring risk for the transmission of AMR.

In contrast, a previous study carried out in other geographical area of Mexico, (Jimenez, Martinez-Urtaza & Chaidez, 2011) reported that all *S.* Anatum isolates recovered from farm animals were susceptible to the antimicrobials tested and their genetic correlation was high. Our data suggested that for *S.* Anatum, only cluster I included three isolates with high genetic similarity and they showed similar MDR profiles.

Similar macrorestriction pulse-typing of *Salmonella* isolates recovered from Mexican food samples have previously allowed the establishment of genetic subgroups within *Salmonella* populations present in the food chain (Wiesner et al., 2009). The genetic diversity detected at a chromosomal level and the discovery of genetic subgroups within *S*. Typhimurum is a valuable tool to monitor the genetic variability within this pathogen, as well as to trace back the development of MDR isolates which could be related to the extensive use of antimicrobials at specific points in the food production chain.

## Conclusions

Multidrug resistance was frequently found among *Salmonella* isolates causing diarrhea in humans, and those obtained from raw beef. Some antimicrobials profiles were shared among isolates occurring in human fecal samples, ground beef and beef carcasses, which suggest possible spreading of the determinants of antimicrobials resistance along the food production chain. The partial matching observed between UPGMA clustering and antimicrobials profiles supports the hypothesis that *Salmonella* is widely distributed ranging from food animals to retail meat products. Additional studies are needed to define if AMR isolates causing salmonellosis in humans in this area of Mexico are originated from foods of animal origin and if the usage of antimicrobial substances as therapeutic agents and growth promoters in cattle is producing selective pressure on the pathogen that increase the risk of having MDR *Salmonella* in foods of animal origin.

##  Supplemental Information

10.7717/peerj.5482/supp-1 Supplemental Information 1 Raw data antimicrobial resistance Final dataset

10.7717/peerj.5482/supp-2 Data S1 PFGE matrix raw data File contains values for characters identified as binary (presence/absence) according to results from PFGE experiments and genetic analysis of the isolates. Columns correspond to number of informative characters. Lines correspond to each isolate analyzed.

## Additional Information and Declarations

### Data Availability

The following information was supplied regarding data availability: The raw data are provided in the Supplemental Files.
